# Preoperative predictors of mortality in intestinal perforation

**DOI:** 10.17305/bb.2025.13309

**Published:** 2025-10-29

**Authors:** Fırat Canlıkarakaya, Serhat Ocaklı, İbrahim Doğan, Nurhak Cihangir Çınkıl, Hüseyin Turap, Cengiz Ceylan

**Affiliations:** 1 Amasya University, Faculty of Medicine, Department of General Surgery, Amasya, Türkiye; 2 Ankara Medipol University, Faculty of Medicine, Department of General Surgery, Ankara, Türkiye; 3 University of Health Sciences Van Education and Research Hospital, Department of General Surgery, Van, Türkiye; 4 Nevşehir Private Cappadocia Hospital, Nevşehir, Türkiye; 5 Amasya Sabuncuoğlu Şerefeddin Training and Research Hospital, Department of General Surgery, Amasya, Türkiye; 6 Eskişehir City Hospital, Department of Gastroinstestinal Surgery, Eskişehir, Türkiye

**Keywords:** Albumin, creatinine, CRP, mortality, bowel perforation

## Abstract

Bowel perforation represents a prevalent and life-threatening emergency within general surgical pathology. This study aims to evaluate clinical and biochemical parameters that predict mortality in cases of bowel perforation. A retrospective analysis was performed on 144 patients who underwent surgical intervention for bowel perforation between 2019 and 2024. Key variables assessed included the albumin/creatinine ratio, age, serum albumin levels, CRP, and history of COVID-19. Mortality-associated variables were analyzed using univariate and multivariate logistic regression, as well as receiver operating characteristic (ROC) analysis. The mean age of the patients was 60 years, with 84 patients (58.3%) being male. The overall mortality rate was 25%. Independent predictors of mortality identified in the study included an albumin/creatinine ratio <3.38 (odds ratio [OR]: 12.666, *P* < 0.001), age >66 years (OR: 3.273, *P* ═ 0.036), and serum albumin levels <3 g/dL (OR: 5.653, *P* ═ 0.002). ROC analysis indicated that the area under the curve (AUC) for the albumin/creatinine ratio was 0.879, establishing it as the parameter with the highest predictive accuracy for mortality. Among patients with a history of COVID-19, ischemia was the predominant cause of perforation (87.5%), while malignancy was the leading cause (41.4%) in those without a COVID-19 history. This difference in etiology was statistically significant (*P* < 0.001). In conclusion, the albumin/creatinine ratio, age, and serum albumin levels are robust parameters for predicting mortality in bowel perforation cases. Furthermore, a history of COVID-19 significantly increases the risk of bowel perforation due to ischemia.

## Introduction

Bowel perforation poses a significant challenge in emergency surgical practice due to its high mortality rates and complex nature. These perforations can occur at any point along the gastrointestinal tract, from the small intestine to the anus, and may result from various etiologies, including ulcers, trauma, inflammatory conditions, or malignancies. Mortality rates ranging from 10% to 40% underscore the severity of the condition and highlight the critical importance of early emergency intervention, which relies on rapid and accurate diagnosis [1, 2].

The management and prognosis of bowel perforation are influenced by multiple factors. The specific etiology of the perforation is crucial in determining the appropriate treatment approach and potential complications. Additionally, the patient’s physiological reserve, which reflects overall health and the ability to endure surgical and recovery stress, significantly impacts outcomes. Existing comorbidities further complicate the clinical scenario and elevate the risk of adverse events. Delays in diagnosis or treatment can lead to severe complications such as sepsis, multiple organ failure, and death, thus underscoring the necessity for prompt recognition and management of this life-threatening condition [3].

The most common causes of bowel perforation include malignancy, ischemia, diverticular disease, and inflammatory bowel disease [2, 4]. These perforations are typically observed in older individuals with comorbid conditions, and mortality risk is heightened in this demographic.

Traditional parameters used to predict mortality generally encompass age, American Society of Anesthesiologists (ASA) score, serum albumin and creatinine levels, and inflammatory markers such as creatinine and C-reactive protein (CRP) [1]. While these indicators provide valuable insights into the patient’s overall health, nutritional status, kidney function, and systemic inflammation, recent studies have highlighted the limitations of relying solely on these individual parameters, as they may not comprehensively reflect the patient’s physiological condition [5].

To address this limitation, there has been a growing emphasis on utilizing composite indicators that simultaneously reflect various systemic influences. This approach aims to facilitate a more comprehensive assessment of patient risk. Next-generation biomarkers, such as the albumin/creatinine ratio, exemplify this trend by offering a more integrated evaluation of the patient’s condition [6]. This specific ratio combines nutritional status (indicated by albumin levels) and kidney function (indicated by creatinine levels), thereby providing a more holistic view of overall health. Such composite markers are increasingly recognized for their potential to enhance risk stratification and improve the accuracy of mortality predictions in clinical settings.

The COVID-19 pandemic has introduced a new paradigm in the etiology of gastrointestinal perforations. SARS-CoV-2 infection has been shown to trigger gastrointestinal complications, including vascular endothelial dysfunction, microthrombosis, and mucosal ischemia [7]. This phenomenon is particularly noted in patients who have recovered from COVID-19, with an increased incidence of ischemia-related perforations.

The literature addressing gastrointestinal perforations beyond peptic ulcers is limited, and comprehensive analyses of mortality predictors are scarce. This study aims to evaluate clinical and biochemical markers that predict mortality in patients undergoing surgical intervention for bowel perforation. The findings are anticipated to contribute to enhanced risk stratification and optimized patient management.

## Materials and methods

**Figure 1.**
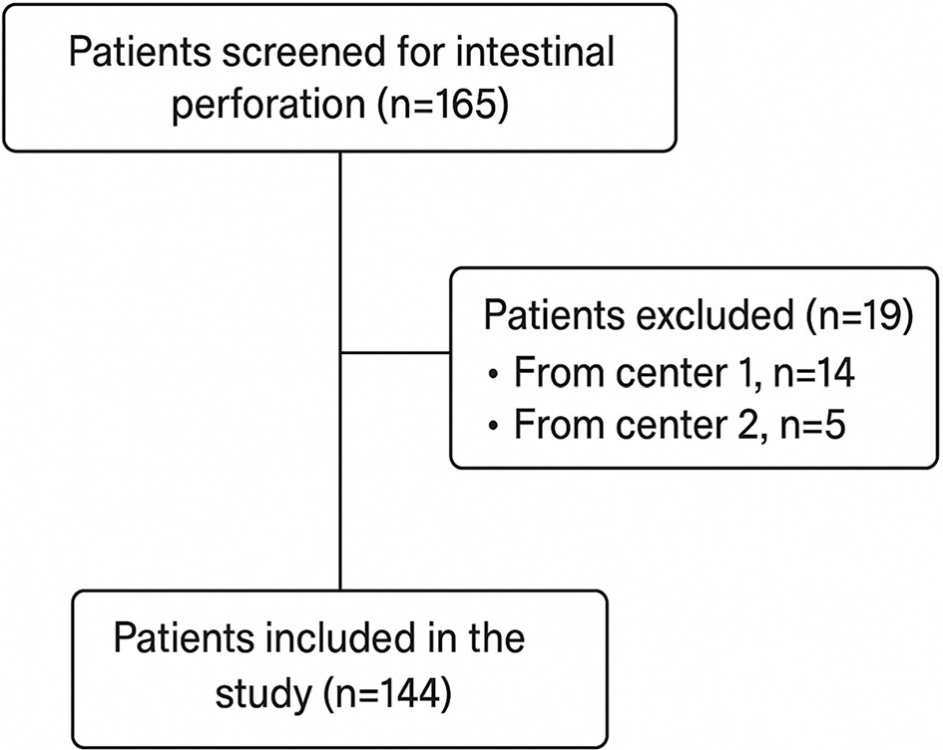
**Flow diagram of patient selection**.

We retrospectively analyzed data from 144 patients who underwent surgery for bowel perforation at two tertiary care centers between January 1, 2019, and December 31, 2024. Following an initial review of 165 cases (*n* ═ 87 from Center 1; *n* ═ 78 from Center 2), 21 patients were excluded due to missing data (14 from Center 1 and 5 from Center 2), yielding the final study cohort (Figure 1). The study received approval from the local ethics committee (Date: 12/06/2025, No: 2025/102). Inclusion criteria consisted of patients over 18 years of age, diagnosed with bowel perforation based on clinical or intraoperative findings, who underwent surgical intervention, and had complete clinical and laboratory data (including CRP, albumin, creatinine, ASA score, and history of COVID-19) along with a minimum of 30 days of postoperative follow-up. COVID-19 was diagnosed using the **Nasopharyngeal Swab Test**. Preoperative laboratory parameters were consistently collected for all patients, utilizing blood parameter values obtained within the final 8 h prior to surgery. Notably, biochemical parameters in both centers were measured using the same analyzer (Siemens Advia Centaur XP). Patients with perforation due to peptic ulcer or a history of chronic kidney disease were excluded from the study.

Demographic data, ASA classifications, comorbidities (including diabetes mellitus, hypertension, chronic obstructive pulmonary disease, coronary artery disease, and history of cerebrovascular events), perforation location, etiology, laboratory values (CRP, albumin, creatinine), and length of hospital stay were obtained from electronic medical records. Mortality was defined as death from any cause occurring within 30 days post-surgery.

### Ethical statement

The study was conducted in accordance with the Declaration of Helsinki and received approval from the local ethics committee (Amasya University Non-Interventional Clinical Research Ethics Committee, Date: 12/06/2025, No: 2025/102).

### Statistical analysis

In this study, sample size and power calculations were performed based on a key predictor of 30-day mortality in patients with intestinal perforation: the Albumin/Creatinine ratio. Multivariate logistic regression analysis revealed a significant association, with an odds ratio (OR) of 12.666 (95% confidence interval [CI]: 4.113–39.004). Sample size and power analyses were conducted using G*Power 3.1 software. The overall mortality rate in the population was estimated at approximately 25%. Power analysis aimed to detect differences in mortality rates between two groups (Alb/Cre < 3.38 vs Alb/Cre ≥ 3.38) at a significance level of α ═ 0.05, with a statistical power of 80% (1-β ═ 0.80). Based on the OR, mortality rates were approximated as 75% (p_1_) in the Alb/Cre < 3.38 group and 25% (p_2_) in the Alb/Cre ≥ 3.38 group. The effect size was quantified using Cohen’s *h* to characterize the difference between these proportions. The power analysis indicated that a minimum total sample size of 111 patients was required to detect this effect. Since the study included 144 patients, the current sample size was deemed sufficient to provide reliable statistical power at the predefined significance and power thresholds. Furthermore, the adequacy of the sample size was supported by receiver operating characteristic (ROC) analysis, where the Albumin/Creatinine ratio demonstrated an area under the curve (AUC) of 0.879 (95% CI: 0.793–0.965), indicating robust predictive accuracy.

The normality of data distribution was assessed using the Kolmogorov–Smirnov test. Nonparametric tests, specifically the Mann–Whitney *U* test, were employed where appropriate. Descriptive statistics, including median and interquartile range (IQR), were reported for continuous variables. Categorical variables were analyzed using the chi-square test, with results presented as frequencies and percentages. Subsequently, a prospective selective multivariate logistic regression analysis was performed to assess variables demonstrating statistical significance in univariate analyses. The goodness of fit of the logistic regression model was evaluated using the Hosmer–Lemeshow test. Statistical significance was defined as *P* < 0.05.

## Results

A total of 144 patients underwent surgical intervention at our clinics for gastrointestinal perforations, excluding those resulting from peptic ulcer disease. The median age of the study population was 60 years (IQR: 41–73.3), with 84 patients (58.3%) being male. Comorbidities included hypertension in 17.4% of patients and coronary artery disease in 13.9%.

According to the ASA physical status classification, 43.1% of patients were classified as ASA II-E, 23.6% as ASA III-E, and 7.6% as ASA IV-E. The most frequent site of perforation was the colon (73.6%), followed by the ileal segments (26.4%).

Etiological analysis revealed malignancy in 38.2% of cases, ischemia in 20.1%, diverticular disease in 17.4%, and inflammatory bowel disease in 8.3%. Postoperative mortality occurred in 36 patients, resulting in a mortality rate of 25% (Table 1).

**Table 1:** Baseline demographic and clinical characteristics of the study population

**Variables**	**Median (IQR)**	**Count (%)**
**Age, years**	60 (41–73.3)	
*Gender*		
Male		84 (58.3%)
Female		60 (41.7%)
*ASA classification*		
ASA IE		37 (25.7%)
ASA IIE		62 (43.1%)
ASA IIE		34 (23.6%)
ASA IVE		11 (7.6%)
*Comorbidities*		
HT		25 (17.4%)
DM		13 (9%)
CAD		20 (13.9%)
COPD		13 (9%)
*Perforation location*		
Colon		106 (73.6%)
Ileum		38 (26.4%)
*Etiology*		
Malignancy		55 (38.2%)
Ischemia		29 (20.1%)
Volvulus		10 (6.9%)
Hernia		13 (9%)
Diverticular disease		25 (17.4%)
IBD		12 (8.3%)
**30-day mortality**		36 (25%)

Abbreviations: IQR: Interquartile range; ASA: American Society of Anesthesiologists Classification; CAD: Coronary artery disease; COPD: Chronic obstructive pulmonary disease; IBD: Inflammatory bowel disease.

**Table 2:** Univariate analysis of preoperative demographic and clinical variables by 30-day mortality status

		**Alive**	**Death**	
**Variables**		**Median (IQR)/*n*(%)**	**Median (IQR)/*n*(%)**	***P* value**
Age, years		54 (36.8–68.5)	74 (65–82)	**<0.001**
Gender	Male	65 (60.2%)	19 (52.8%)	0.435
ASA classification	ASAIE	36 (33.3%)	1 (2.8%)	**<0.001**
	ASAIIE	54 (50%)	8 (22.2%)	
	ASAIIIE	18 (16.7%)	16 (44.4%)	
	ASAIVE	0 (0%)	11 (30.6%)	
HT		17 (15.7%)	8 (22.2%)	0.374
DM		11 (10.2%)	2 (5.6%)	0.401
CAD		12 (11.1%)	8 (22.2%)	0.095
COPD		9 (8.3%)	4 (11.1%)	0.615
COVID-19 history		12 (11.1%)	4 (11.1%)	1
Perforation location	Colon	77 (71.3%)	29 (80.6%)	0.275
	Ileum	31 (28.7%)	7 (19.4%)	
Etiology	Malignancy	40 (37%)	15 (41.7%)	0.054
	Ischemia	17 (15.7%)	12 (33.3%)	
	Volvulus	7 (6.5%)	3 (8.3%)	
	Hernia	13 (12%)	0 (0%)	
	Diverticular disease	20 (18.5%)	5 (13.9%)	
	IBD	11 (10.2%)	1 (2.8%)	
Albumin, g/dL		3.34 (2.99-3.63)	2.4 (2.03-2.88)	**<0.001**
Creatinine, mg/dL		0.72 (0.59-0.91)	1.78 (1.01-2.98)	**<0.001**
CRP, mg/L		70 (25.3-158)	152.81 (94.5-269)	**<0.001**
Albumin/creatinine		4.53 (3.71-5.64)	1.4 (0.76-2.45)	**<0.001**

Note: *P* value < 0.05 was considered statistically significant. Abbreviations: IQR: Interquartile range; ASA: American Society of Anesthesiologists Classification; HT: Hypertension; DM: Diabetes mellitus; CAD: Coronary artery disease; COPD: Chronic obstructive pulmonary disease; IBD: Inflammatory bowel disease; CRP: C-reactive protein.

In univariate analyses, several variables were significantly associated with mortality, including age (54 [IQR: 36.8–68.5] years vs 74 [IQR: 65-82] years, *P* < 0.001), ASA score (*P* < 0.001), serum albumin (3.34 [IQR: 2.99–3.63] g/dL vs 2.4 [IQR: 2.03–2.88] g/dL, *P* < 0.001), creatinine (0.72 [IQR: 0.59–0.91] mg/dL vs 1.78 [IQR: 2.03–2.88] mg/dL, *P* < 0.001), CRP (70 [IQR: 25.3–158] mg/L vs 152.81 [IQR: 94.5–269] mg/L, *P* < 0.001), and the albumin-to-creatinine ratio (ACR) (4.53 [IQR: 3.71–5.64] vs 1.4 [IQR: 0.76–2.45], *P* < 0.001) (Table 2).

ROC analysis was conducted to evaluate the predictive power of various variables for mortality. An age greater than 66 years was associated with an area under the curve (AUC) of 0.764 (95% CI: 0.671–0.856, *P* < 0.001). Serum albumin levels below 3 g/dL demonstrated a strong discriminatory ability, with an AUC of 0.845 (95% CI: 0.769–0.922, *P* < 0.001). A CRP level greater than 122 mg/L presented an AUC of 0.696 (95% CI: 0.600–0.792, *P* < 0.001). Notably, an ACR below 3.38 exhibited the highest predictive accuracy, with an AUC of 0.879 (95% CI: 0.793–0.965, *P* < 0.001) (Table 3).

In the multivariate logistic regression analysis, several independent predictors of mortality were identified. Age greater than 66 years was associated with a significantly increased risk of mortality (OR: 3.273, 95% CI: 1.081–9.905, *P* ═ 0.036). Serum albumin levels below 3 g/dL also emerged as an independent risk factor (OR: 5.653, 95% CI: 1.849–17.287, *P* ═ 0.002). Although CRP levels greater than 122 mg/L indicated an increased OR, this association was not statistically significant (OR: 1.392, 95% CI: 0.462–4.194, *P* ═ 0.556). Importantly, an ACR less than 3.38 was identified as the strongest independent predictor of mortality (OR: 12.666, 95% CI: 4.113–39.004, *P* < 0.001) (Table 4).

An analysis was also performed to assess the relationship between a history of COVID-19 infection and the etiology of luminal gastrointestinal perforations (Table 5). Among patients without a history of COVID-19, malignancy was the most common etiology (41.4%), followed by diverticular disease (19.5%), inflammatory bowel disease (9.4%), hernia (10.2%), and volvulus (7.8%). In contrast, among patients with a history of COVID-19, ischemia was the predominant etiology, observed in 87.5% of cases. Only 12.5% of patients with a history of COVID-19 had malignancy as the underlying cause, and no cases of diverticular disease, inflammatory bowel disease, hernia, or volvulus were recorded in this group. This difference in distribution was statistically significant (*P* < 0.001), indicating a strong association between prior COVID-19 infection and the ischemic etiology of gastrointestinal perforation.

**Table 3:** Diagnostic performance of preoperative predictors for 30-day mortality based on ROC analysis

**Variable**	**AUC**	**95% CI**	**Sensitivity**	**Specificity**	**PPV**	**NPV**	***P* value**
Age >66 year	0.764	0.671–0.856	75%	69.44%	45%	89.29%	**<0.001**
Alb <3 g/dL	0.845	0.769–0.922	75%	83.3%	60%	90.91%	**<0.001**
CRP >122 mg/L	0.696	0.6–0.792	72.2%	64.49%	40.62%	87.34%	**<0.001**
Alb/Cre <3.38	0.879	0.793–0.965	75%	96.3%	87.1%	92.04%	**<0.001**

Note: Sensitivity, specificity, PPV, and NPV values are calculated based on the optimal cut-off points determined via Youden Index. All variables were dichotomized at these thresholds for analysis. Units: Albumin in g/dL, CRP in mg/L. Albumin/Creatinine is a unitless ratio calculated using albumin (g/dL) and creatinine (mg/dL). Abbreviations: AUC: Area under the curve; CI: Confidence interval; PPV: Positive predictive value; NPV: Negative predictive value.

**Table 4:** Multivariate analysis

**Variable**	**OR**	**95% CI**	***P* value**
Age >66 year	3.273	1.081–9.905	**0.036**
Albumin <3 g/dL	5.653	1.849–17.287	**0.002**
CRP >122 mg/L	1.392	0.462–4.194	0.556
Albumin/Creatinine <3.38	12.666	4.113–39.004	**<0.001**

Note: *P* value of <0.05 was considered statistically significant. Abbreviations: CRP: C-reactive protein; OR: Odds ratio; CI: Confidence interval.

**Table 5:** Analysis of the relationship between the history of Covid-19 and the etiology of luminal organ perforation

**Covid-19 history etiology**	**Absence count (%)**	**Presence count (%)**	***P* value**
Malignancy	53 (41.4%)	2 (12.5%)	**<0.001**
Ischemia	15 (11.7%)	14 (87.5%)	
Other	60 (46.9%)	0 (0%)	

*Other pathologies include volvulus, hernia, diverticular disease, and inflammatory bowel diseases. *P* value of <0.05 was considered statistically significant.

## Discussion

Bowel perforations represent critical clinical conditions necessitating emergency surgical intervention, often associated with elevated mortality rates. This study examined parameters predicting mortality in bowel perforations and established that a low ACR, advanced age, and low albumin levels are independent risk factors.

Recent studies have identified the ACR as a biomarker reflecting physiological reserve in sepsis and acute surgical patients. This ratio serves as a multidimensional risk indicator, simultaneously reflecting nutritional status and renal function. Lin et al. [8] reported that the ACR inversely correlates with 28-day mortality in septic ICU patients. Their research indicated that as the ratio decreases, the risk of mortality significantly increases, highlighting a nonlinear relationship. In our study, an ACR <3.38 emerged as the parameter with the highest predictive accuracy for mortality (AUC: 0.879). This finding suggests that the ratio may serve as a valid prognostic tool in acute surgical conditions such as bowel perforation.

We hypothesize that the predictive capacity of the ACR for mortality can facilitate effective surgical triage, aiding in decision-making for allocating patients to either damage control surgery (DCS) or definitive surgical repair.

Low albumin levels have long been recognized as a marker of poor prognosis in surgical patients. Albumin is crucial for critically ill patients due to its anti-inflammatory effects, antioxidant properties, and regulation of vascular permeability. Vincent et al. [9] demonstrated a strong association between hypoalbuminemia and mortality in acute illnesses. Sung et al. [1] reported a 100% mortality rate in patients with an albumin level ≤2 g/dL, a postoperative sequential organ failure assessment (p-SOFA) score ≥7, and body temperature ≤36 ^∘^C. Although albumin alone is not an independent predictor, it is one of the three important parameters. In our study, an albumin level <3 g/dL was identified as an independent predictor of mortality (OR: 5.653). These findings suggest that albumin provides information about not only nutritional status but also systemic inflammation and organ function.

Advanced age is a well-established risk factor for mortality in cases of bowel perforation. As individuals age, reduced physiological reserve, a higher prevalence of comorbidities, and a diminished immune response impair their ability to withstand surgical stress. Yan et al. [3] reported a significant correlation between age and mortality among patients undergoing surgery for gastrointestinal perforation. In the current study, age greater than 66 was identified as an independent risk factor for mortality (OR: 3.273). This finding underscores that age serves not merely as a demographic variable but also as an indicator of physiological resilience.

CRP is one of the most prevalent biochemical markers of systemic inflammation and acts as an acute-phase reactant. Its levels typically rise significantly in various acute surgical conditions, such as sepsis, peritonitis, and gastrointestinal perforations. However, the predictive value of CRP for mortality can vary based on its timing, the presence of organ dysfunction, and the patient’s physiological reserve. In this study, a CRP level exceeding 122 mg/L was significantly associated with mortality; however, it was not an independent predictor in multivariate analysis. This suggests that CRP alone is insufficient as a prognostic marker and should be assessed in conjunction with other parameters. In patients with peritonitis, especially those in high-risk situations like gastrointestinal perforation, CRP levels greater than 150 mg/L have been linked to increased mortality [1]. Çakır et al. [10] compared CRP, albumin, and the CRP/albumin ratio in septic patients, finding that the CRP/albumin ratio is a stronger predictor of mortality. This ratio provides a more comprehensive risk assessment, reflecting both inflammatory burden and nutritional status. While CRP levels alone may not serve as an adequate prognostic tool in acute surgical conditions such as bowel perforation, they can significantly aid clinical decision-making when analyzed alongside factors like albumin, creatinine, and age.

Malignancy-related perforations are associated with high mortality rates, often due to delayed diagnosis, immunosuppression, and poor overall condition. Wu et al. [2] reported a 19.2% mortality rate in small bowel perforations caused by malignancy. In this study, malignancy was identified as the most common etiological factor, but its direct association with mortality was not statistically significant. This indicates that etiology alone does not dictate mortality; rather, the patient’s overall condition, physiological reserve, and other contributing factors play a more critical role.

SARS-CoV-2 enters cells via ACE2 receptors, which are widely expressed not only in the lungs but also throughout the gastrointestinal tract, including the esophagus, stomach, small intestine, and colon. Viral binding to endothelial cells can initiate processes such as microvascular thrombosis, endothelial dysfunction, and hypercoagulability. This can particularly result in non-occlusive ischemia in the mesenteric circulation, leading to necrosis and perforation of the gastrointestinal tract wall. A large-scale epidemiological study conducted in Japan by Okura et al. [11] analyzed the incidence of gastrointestinal tract perforation in 276 patients diagnosed with COVID-19. The study found that the risk of developing bowel perforation within the first week after COVID-19 diagnosis was 6.62 times higher.

Sarkardeh et al. [12] conducted a multicenter case series involving 24 patients who developed simultaneous bowel ischemia alongside COVID-19. In 87.5% of these patients, bowel necrosis and perforation occurred without macrovascular mesenteric ischemia. In our study, ischemia was the most common cause of perforation in patients with a history of COVID-19 (87.5%), while malignancy was the leading cause in those without COVID-19 (41.4%). This difference in distribution was statistically significant, suggesting that COVID-19 affects the gastrointestinal system not only symptomatically but also at structural and vascular levels.

## Conclusion

In conclusion, the findings of this study identified the preoperative albumin/creatinine ratio, age, and albumin level as independent risk factors for mortality. Incorporating these parameters into clinical decision-making could enhance risk stratification and optimize patient management. Furthermore, the increased incidence of ischemia-related perforations in patients with a history of COVID-19 underscores the need for special consideration of this patient population. Future studies should prospectively validate these parameters and explore their integration into risk scoring systems.

## Data Availability

Due to patient confidentiality and the protection of personal data, the datasets of this study are not publicly available. However, the data may be shared upon reasonable request to the corresponding author, provided that all patient identifiers and personal information remain confidential.
